# Volumetric Analysis of Amygdala, Hippocampus, and Prefrontal Cortex in Therapy-Naive PTSD Participants

**DOI:** 10.1155/2014/968495

**Published:** 2014-03-13

**Authors:** Ana Starcevic, Srdjan Postic, Zoran Radojicic, Branislav Starcevic, Srdjan Milovanovic, Andrej Ilankovic, Ivan Dimitrijevic, Aleksandar Damjanovic, Milan Aksić, Vidosava Radonjic

**Affiliations:** ^1^Institute of Anatomy “NikoMiljanić”, Medical Faculty, Dr Subotića 4, University of Belgrade, Belgrade, Serbia; ^2^Clinic of Dental Prosthetic, The Faculty of Stomatology, University of Belgrade, Serbia; ^3^Institute for Statistics, Faculty of Organizational Sciences, University of Belgrade, Serbia; ^4^Institute for Traumatology, Emergency Center, Clinical Center of Serbia, Medical Faculty, University of Belgrade, Serbia; ^5^Institute for Psychiatry, Clinical Center of Serbia, Medical Faculty, University of Belgrade, Serbia

## Abstract

*Objective*. In our study we have hypothesized that volume changes of amygdala, hippocampus, and prefrontal cortex are more pronounced in male posttraumatic stress disorder participants. *Material and Methods*. We have conducted a study of 79 male participants who underwent MRI brain scanning. PTSD diagnosis was confirmed in 49 participants. After MRI was taken all scans were software based volume computed and statistically processed. *Results*. We found that left amygdala is the most significant parameter for distinction between PTSD participants and participants without PTSD. There were no significant differences in volumes of hippocampi and prefrontal cortices. Roc curve method outlined left amygdala AUC = 0.898 (95% CI = 0.830–0.967) and right amygdala AUC = 0.882 (95% CI = 0.810–0.954) in the group of PTSD participants which makes both variables highly statistically significant. *Conclusion*. The present investigation revealed significant volume decrease of left amygdala in PTSD patients. Concerning important functions of the amygdala and her neuroanatomical connections with other brain structures, we need to increase number of participants to clarify the correlation between impared amygdala and possible other different brain structures in participants with PTSD.

## 1. Introduction

Posttraumatic stress disorder or PTSD is an anxiety disorder that can occur following the experience or witnessing of traumatic event [1, 2]. Participants with migraine or tension headaches report high rates of exposure to traumatic events. In addition, about 17% have symptoms consistent with a PTSD diagnosis [3–7]. MRI guided studies revealed reduction of the limbic structures of the brain: hippocampus is believed to be the most frequently reduced structure [8–10], but also anterior cingulate cortex and amygdala are reported as the structures that undergo the volume changes in PTSD [11–17]. Despite some inconsistencies in findings, there is currently no clear evidence for abnormal amygdala volumes in PTSD. One study reported smaller amygdala volumes in a cohort of breast cancer survivors with intrusive recollections compared to survivors without intrusive recollections, but none of the participants met diagnostic criteria for PTSD [18]. Volumes of caudate nucleus, putamen, and lateral ventricles were found to decrease bilaterally in PTSD participants with headaches [19].

In the present study, we have hypothesized that volume changes in amygdala, hippocampus, and prefrontal cortex are more pronounced in male therapy-naive PTSD participants with headaches.

## 2. Materials and Methods

We have included 79 male participants from Psychiatric Hospital Clinical Centre, Serbia, and Psychiatric Clinic “Katarza.” Recruiting criteria for this study were

1. absence of comorbidity of any kind of psychiatric disorders or diseases;
2. absence of comorbidity of any kind of neurological disorders or diseases;
3. absence of previous psychotropic medication;
4. absence of alcohol or substance abuse;
5. no data of psychiatric illnesses, homicides, or suicides in participants family anamnesis;
6. absence of memory impairment;
7. absence of any kind of head trauma.

Excluding criteria were

1. the traces of the psychoactive substances in the urine and blood;
2. major brain abnormalities detected on MRI scans;
3. evidence about the personality disorder.

Structured psychiatric interview was taken from all participants and they went for psychological testing and magnetic resonance imaging. All participants were followed up for a one-month period and were advised to undergo psychotherapy without taking any medication. They all underwent biochemical testing for illicit drugs. Diagnosis of disorder was assessed according to the guidelines in the 10th revision of the International Classification of Diseases (ICD-10). They were also told to record how often they had headaches and to mark on the pain rating scale when the strongest pain occurred during the day. The scale pain has ten segments, ranging from 0 (no pain) to 10 (the worst possible pain). Hamilton depression rating scale [20] was used for rating the depression level. After we collected all the data from the examination, the initial group of 79 subjects was divided into two groups. First group consisted of 49 participants with PTSD diagnosis and the second consisted of 30 participants without PTSD diagnosis. From the total amount of 79 participants, headache as a symptom appeared in 39 participants. Twenty-five of them (31.6%) had headaches at least twice a week. Fourteen participants (17.7%) had less than twice-a-week headaches and ten participants reported no headaches at all. We found that 49 participants (62%) were with PTSD diagnosis and 30 participants (38%) without PTSD diagnosis. In the range of Hamilton's scale of depression, from the total amount of 79 participants we found 33 participants (41.8%) with minor depression.

We included only males because of potential neuroendocrine involvement on headaches in females. The study was conducted in the period from June to February 2012. All participants signed a written consent.

## 3. Magnetic Resonance Imaging

This MRI study was performed using a 3.0 T whole body MRI scanner (Philips Medical Systems, Best, The Netherlands). After the scanning, all participants were coded in order to blind the volumetric evaluation team and sent for the subsequent volumetric analysis.

Volume measurements of the prefrontal cortex and hippocampus were performed on 3D T 1-weighted MR images (acquisition parameters were as follows: TR = 9.8 ms; TE = 4.6 ms; flip angle = 8; section thickness = 1.2 mm; number of sections = 120; no section gap; whole brain coverage; FOV = 224 mm; matrix = 192; reconstruction matrix = 256). Routine T 2-weighted MRI and FLAIR were performed to rule out a mass lesion as a contributory factor to memory loss or cognitive decline [21–23].

During registration, the input data (3D T1 images) were transformed to 152 standard spaces, by means of transformations based on 12 degrees of freedom (i.e., three translations, three rotations, three scalings, and three skews). After subcortical and cortical registration (MNI 152 space), a mask was applied to locate the cortical and subcortical structures of interest, followed by segmentation based on shape models and voxel intensities [24]. The absolute volumes of structures obtained were calculated, taking into account the transformations made in the first stage [21, 25]. Finally, a boundary correction was used to determine whether boundary voxels belonged to the structure examined or not. In this study, a* Z*-value of 3 was used, corresponding to a 99.998% certainty that the voxels belonged to the particular structure. After registration and segmentation of all 139 MR scans, all segmented the regions of interest were visually checked for errors in registration and segmentation. Brain tissue volume was estimated with MIPAV software package, Medical Image Processing, Analysis, and Visualization (National Institute of Health, Bethesda, USA).

## 4. Results

There was no correlation between PTSD participants and associated parameters except in case of left and right amygdala. The same is with participants without PTSD where we did not find any correlation between hippocampi and prefrontal cortices. We found no difference in the parameters and observed groups and the same parameters.

Using the* t*-test for the independent samples, we found that both left (*t* = 8.453, df = 72.700; *P* < 0.001) and right (*t* = 8.228; df = 68.675; *P* < 0.001) amygdala volumes have statistically significant difference between PTSD participants and participants without PTSD (Table 1).

We found that left amygdala is the most significant parameter (Wald = 16.476; df = 1; *P* < 0.001) for distinction between PTSD and participants without PTSD, using multivariate logistic regression forward method (Table 2).

The receiver operating characteristic (ROC) curve is the plot that displays the full picture of trade-off between the sensitivity and across a series of cut-off points. Area under the Roc curve is considered as an effective measure of inherent validity of a diagnostic test. This curve is useful in evaluating the discriminatory ability of a test to correctly pick up diseased and nondiseased subjects or find optimal cut-off point to the least misclassify diseased and nondiseased subjects.

**Figure 1:**
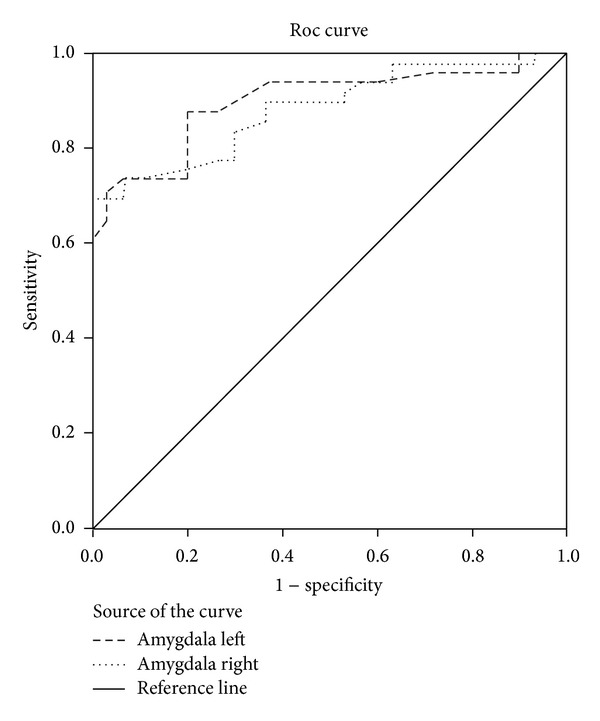
Roc curve method outlined left amygdala AUC = 0.898 (95% CI = 0.830–0.967) and right amygdala AUC = 0.882 (95% CI = 0.810–0.954). Both variables are statistically significant.

Roc curve method outlined left amygdala AUC = 0.898 (95% CI = 0.830–0.967) and right amygdala AUC = 0.882 (95% CI = 0.810–0.954) in the group of PTSD participants which makes both variables very statistically significant (Figure 1). Correlation between left and right amygdala in the group of PTSD participants was *r* = 0.878; *P* < 0.001. The same correlation is with participants without PTSD *r* = 0.830; *P* < 0.001. There was no difference between these two groups *P* > 0.05.

The comparison to the cut-off point shows 81.6% sensitivity and 80% specificity, which means that left amygdala can clearly indicate who is PTSD participant and who is not, *P* < 0.001. The classification of up to and above the limit of 1.87 indicates that it can be used as the test considering that OR = 17.778 with 95% CI of 5.627–56.162; that is, those with lower left amygdala score are in the relative risk of 4.082 with 95% CI of 1.971–8.452.

Additionally, the limit for the right amygdala is 1.88, because different scores were obtained only for amygdala. We noticed the association between the group of participants without PTSD and PTSD participants and the right amygdala scores of up to and above 1.88. In this case, the sensitivity of 77.6% and specificity of 73.3% are observed, which is less compared to the left amygdala but still statistically significant *P* < 0.001.

Results for right amygdala that showed statistical significance are OR = 9.500 with 95% CI from 3.320–27.181 and the relative risk of those with scores lower than 1.88 shows that the RR = 2.908 (1.577–5.346).

We used positive/negative ratio for finding cut-off point of the following variables: for left amygdala it is 1.87 and for right amygdala it is 1.88. Those numbers divide initial group of 79 participants in two groups on those with PTSD diagnosis (PTSD participants) and participants without PTSD (Table 3).

## 5. Discussion

In our study we compared volumes of amygdala, hippocampus, and prefrontal cortex in male PTSD participants who were therapy-naive and participants without PTSD. Headaches occurred in 39 participants (49.3%). Several recent publications have emphasized the relationship between life stressors and recurrent headaches. According to de Leeuw et al. [5], almost 64% of the headache participants reported one or more major traumatic stressors. It is not entirely clear why people with PTSD may be more likely to experience problems with headaches. However, stress has been linked to the occurrence of headaches, and the symptoms of PTSD can definitely contribute to very high levels of stress and emotional strain. In addition, headache patients tend to have more stressful events in their daily lives. PTSD can greatly interfere with many aspects of a person's life, such as at work and in relationships. This is likely going to cause more stress, increasing the likelihood of headaches. In some cases, the type of traumatic event a person with PTSD has experienced may increase the likelihood of headaches. There are several potential mechanisms for the association between PTSD and migraine. Potential mechanisms include dysfunction of the central monoaminergic system and the hypothalamic-pituitary-adrenal (HPA) axis. It is unknown why the PTSD-migraine association is stronger in men than women. Genetic sex differences and sex differences in the stress response of the HPA axis may play a role [26]. Trauma survivors who have PTSD may have trouble with their close family relationships or friendships. Their symptoms can cause problems with trust, closeness, communication, and problem solving, which may affect the way the survivor acts with others. In turn, the way a loved one responds to him or her affects the trauma survivor. A circular pattern may develop that could harm relationships. That can lead to anxiety, general anxiety disorder and depression. In our investigation, from the total amount of 79 participants, in the range of Hamilton's scale of depression, we found 33 participants (41.8%) with minor depression.

Our analysis showed hippocampal decrease consistent with the finding of Kitayama et al., [9]. Karl et al. [8] showed significantly smaller hippocampal volumes in persons with PTSD compared to controls with and without trauma exposure. Smith [10] conducted a study in which on average PTSD participants had a 6.9% smaller left hippocampal volume and a 6.6% smaller right hippocampal volume compared with control subjects. Ahmed et al., [27] noted decreased volumes of the prefrontal cortex in their MRI study. We did not find any correlation between hippocampi and prefrontal cortices.

In our investigation of volumes of amygdala, hippocampi, and prefrontal cortices, the interesting finding is that the left amygdala was significantly smaller than right. Gurvits et al., [28] found greater right amygdala volume in their meta-analysis, and Wignall et al., [29] showed postmortem asymmetry in amygdala in healthy adult human brains. Woon and Hedges [30] found no significant volume differences in the left and right amygdala in comparing subjects with PTSD both with healthy comparison subjects not exposed to trauma and with healthy, trauma-exposed subjects without PTSD.

Although most previous studies reported nonsignificant findings, some studies showed a trend towards significance for smaller amygdala volumes in PTSD [31–34]. The investigations of PTSD and amygdala volume with the largest samples included PTSD groups with *n* = 4443 and *n* = 2844 but were conducted in children and therefore do not generalize well to adults due to developmental changes in brain structure and connectivity. All studies of adults had a sample size of fewer than 20 in the PTSD group, with the majority of studies having 15 or fewer participants [22, 23, 25, 30, 35]. Consistent with our findings and relevant to the core symptom cluster of reexperiencing symptoms in PTSD, smaller amygdala volume was associated with the presence of cancer-related intrusive recollections in a sample of 76 breast cancer survivors [3]. It is worthwhile to consider the factors that may have produced a significant association of decreased amygdala volume given the preponderance of negative findings in prior studies. The small effect sizes observed from the meta-analyses suggest that they were underpowered to detect significant differences. Normal amygdala volumes do not necessarily preclude functional abnormalities in the amygdala in participants with PTSD. As a case in point, the results of a functional neuroimaging meta-analysis in participants with PTSD found evidence of amygdala abnormalities, particularly in the left amygdala, where two distinct clusters of abnormal function were identified: a ventral anterior hyperactivation cluster and a dorsal posterior hypoactivation cluster [34]. The distinct functional expression within the subregions of the amygdala highlights the need for more focused studies of PTSD with high-resolution structural MRI technology.

## 6. Conclusion

Our results provide evidence of an association between a smaller amygdala, hippocampus, and prefrontal cortices volumes and PTSD therapy-naive participants suffering from headaches. The headaches that occur at least twice a week might be possible sign of the decrease in volumes of amygdala. Results of our MRI research study provide us significant findings which can contribute to better understanding of the neuroanatomical substrate of PTSD, but the increase of the number of participants to clarify further significance of changed volumes of different brain structures in participants with PTSD, as well as the establishment of a reliable animal model, is mandatory.

## Figures and Tables

**Table 1:** *t*-test (other parameters showed no statistically significant differences).

Parameters		*N*	Mean	Std. Deviation	*t*	df	*P*
Age	PTSD+	49	46.4694	8.16523	−0.208	77	0.836
	PTPD−	30	46.8667	8.39020			
Left hippocampus	PTSD+	49	3.5616	0.71178	−0.965	55.577	0.339
	PTPD−	30	3.6637	0.15949			
Right hippocampus	PTSD+	49	3.5704	0.70175	−1.200	55.774	0.235
	PTPD−	30	3.6957	0.15939			
Left amygdala	PTSD+	49	1.6645	0.20737	−8.453	72.700	<0.001
	PTPD−	30	1.9553	0.09587			
Right amygdala	PTSD+	49	1.6760	0.22086	−8.228	68.675	<0.001
	PTPD−	30	1.9677	0.08854			
Left prefrontal cortex	PTSD+	49	164.7553	21.71777	0.445	77	0.657
	PTPD−	30	162.4967	22.14106			
Right prefrontal cortex	PTSD+	49	162.2963	21.39362	0.445	77	0.657
	PTPD−	30	160.0714	21.81059			

**Table 2:** Left amygdala is shown to be important parameter.

Variables in the equation		Wald	df	Sig.
Step 1	Amygdala left	16.476	1	<0.001
	Constant	16.419	1	<0.001

**Table 3:** In the prediction to the specified limits of up to 1.88 and 1.87 can be significant, primarily those for amygdala left (*P* = 0.007) while those for amygdala right show not statistical significance (*P* = 0.822) with the Nagelkerke *R* Square of 0.438, which is significant.

	Wald	df	Sig.	HR	95% C.I. for HR	
					Lower	Upper
Amygdala left on cut-off point 1.87	7.177	1	0.007	22.200	2.298	214.443
Amygdala right on cut-off point 1.88	0.051	1	0.822	0.771	0.081	7.384
Constant	18.098	1	<0.001	0.152		
